# Post-polio Syndrome: More Than Just a Lower Motor Neuron Disease

**DOI:** 10.3389/fneur.2019.00773

**Published:** 2019-07-16

**Authors:** Stacey Li Hi Shing, Rangariroyashe H. Chipika, Eoin Finegan, Deirdre Murray, Orla Hardiman, Peter Bede

**Affiliations:** Computational Neuroimaging Group, Academic Unit of Neurology, Biomedical Sciences Institute, Trinity College Dublin, Dublin, Ireland

**Keywords:** postpolio syndrome, PPS, polio, poliomyelitis, neuroimaging, biomarker, clinical trials, motor neuron disease

## Abstract

Post-polio syndrome (PPS) is a neurological condition that affects polio survivors decades after their initial infection. Despite its high prevalence, the etiology of PPS remains elusive, mechanisms of progression are poorly understood, and the condition is notoriously under-researched. While motor dysfunction is a hallmark feature of the condition, generalized fatigue, sleep disturbance, decreased endurance, neuropsychological deficits, sensory symptoms, and chronic pain are also often reported and have considerable quality of life implications in PPS. The non-motor aspects of PPS are particularly challenging to evaluate, quantify, and treat. Generalized fatigue is one of the most distressing symptoms of PPS and is likely to be multifactorial due to weight-gain, respiratory compromise, poor sleep, and polypharmacy. No validated diagnostic, monitoring, or prognostic markers have been developed in PPS to date and the mainstay of therapy centers on symptomatic relief and individualized rehabilitation strategies such as energy conservation and muscle strengthening exercise regimes. Despite a number of large clinical trials in PPS, no effective disease-modifying pharmacological treatments are currently available.

## Introduction

Poliomyelitis was one of the most acutely debilitating infections of the twentieth century that affected millions in the 1940 and 1950s and more recently in India during an outbreak in 1988 (1). Following the introduction of the polio vaccine in the mid-1950s and early 1960s, there has been a dramatic decline in the number of new polio cases and it is estimated to be 99% eradicated today. Despite the enormous progress in the eradication of the polio virus, 15–20 million people across the world still suffer from the sequelae of the infection (2). A large proportion of polio survivors has been presenting with a constellation of new neurological symptoms that has been described as Post-Polio Syndrome (PPS). The description of PPS is attributed to Jean-Martin Charcot in 1875 but was only widely recognized by the medical community in the early 1980s (3). PPS is characterized by new neurological deficits after a long period of neurological stability, typically at least 15 years after the initial polio infection. PPS may manifest as new, persistent, and progressive muscle weakness, atrophy, limb fatigability, myalgia, arthralgia, and dysphagia, but also as generalized fatigue, which typically has a considerable impact on the patients' quality of life. The estimates of the percentage of polio patients affected by PPS are inconsistent, varying between 20 and 85% (4, 5) depending on the diagnostic criteria applied (2). As a result, despite the rarity of acute polio infection in the modern world, PPS is likely to persist for the next few decades. Despite its prevalence, post-polio syndrome remains surprisingly under-researched and poorly characterized. The purpose of this review is to provide a comprehensive overview of the aetiological, genetic, diagnostic, prognostic factors, and treatment modalities in PPS while highlighting key gaps that require further research.

## Methods

A literature search was performed on PubMed using the search term “post-polio syndrome,” “postpolio syndrome” or “post-polio syndrome” alone and in combination with “epidemiology,” “pathophysiology,” “clinical features,” “fatigue,” “neurophysiology,” “brain imaging,” “electromyography,” “inflammation,” “diagnosis,” “management,” “clinical trial,” “longitudinal,” “cross-sectional,” “case report,” “autopsy,” and “post mortem.” Only articles written in English and published between January 1980 and May 2019 were selected for literature review. Identified publications were categorized into “academic” papers discussing pathophysiology, genetic susceptibility, biology, and “clinical” papers focusing on diagnostic criteria, management, rehabilitation, and clinical trials.

## Results

### Pathophysiology

During the acute poliomyelitis infection, 95% of those infected remain asymptomatic or only suffer flu-like symptoms while the remaining 5% succumb to the paralytic form of the disease. Acute poliomyelitis is typically spinal, affecting the limbs and respiratory musculature, but bulbar manifestations affecting speech and swallow are also well-documented. Polioenterovirus type 1 is the main cause of meningeal, spinal cord and brain inflammation as it can cross the blood-brain barrier independently from poliovirus receptors (6, 7). Ensuing anterior horn degeneration, and apoptosis post infection has been widely recognized as the hallmark feature of paralytic poliomyelitis. Following the acute phase, axonal sprouting takes place reinnervating the muscle of the affected regions (8, 9). Motor units gradually become abnormally enlarged, up to 7-fold their original size (10) rendering them metabolically unsustainable (11). This process can take up to three decades from the acute infection to the development of PPS symptoms (12). The concomitant denervation-reinnervation process is evidenced by electromyography (EMG) findings (13–17) and muscle histology showing small angulated fibers (18, 19) and muscle fiber type-grouping (15). Metabolic stress (11, 20), overuse (21, 22), physiological aging (20, 23), and persistent inflammation (24) are also thought to contribute to gradual motor unit failure. Motor units loss has been consistently correlated to functional decline in longitudinal studies (13, 14, 25, 26). Overuse of functioning muscle units is thought to induce detrimental structural alterations (27, 28). Cellular adaptation in the muscles, such as fiber alteration from type II (fast) to type I (slow) (28), changes in contractile properties (29–31), and muscle hypertrophy (9) are likely to contribute to muscular fatigue and myalgia in PPS. The persistence or reactivation of polio virus in polio survivors has also been suggested with conflicting reports. Two research studies (7, 32) have identified polio-virus (PV) genomic sequences in the CSF and peripheral leucocytes as well as high serum IgM anti-PV antibody titres, which were absent in stable polio survivors and in other neurodegenerative groups (33). Other studies however could not confirm these findings (34). An inflammatory or autoimmune basis to post-polio syndrome has also been proposed. This hypothesis originates from post mortem observations of inflammatory changes in the spinal cord of PPS patients (35, 36). The role of inflammation is also supported by *in vivo* evidence. Increased serum and CSF levels of pro-inflammatory cytokines and peptides such as TNF-α, IFN-γ were repeatedly observed in PPS (37–39). Furthermore, TNF-α and IFN-γ levels respond to IVIg therapy in PPS, and remain unchanged in controls (37, 38, 40). However, no correlations have been detected between symptom severity (38), rate of decline (37), and pro-inflammatory peptide levels. Skeletal muscle biopsies also exhibit inflammatory changes and increased expression of prostaglandin E2 synthetic pathway enzymes (41). Relatively limited evidence exists to support the autoimmune basis of PPS. One study identified high titres of PV antibodies concurrently with high levels of regulatory T cells (42), while another study (43) found normal levels of immune complexes in PPS patients. No specific anti-muscle or anti-neuronal autoantibodies have been associated with PPS (44). A genetic predisposition for PPS has also been investigated, but no conclusive risk profile has been identified to date. *SMN* gene deletion (45, 46) associated with spinal muscular atrophy (SMA) was not reported in PPS, but Fc-gamma receptor IIIA polymorphisms may play a role in the predisposition to PPS (47).

### Neuropathology and Neuroimaging

Post-mortem studies are conflicting with regards to cerebral involvement in post-polio syndrome. Post-mortem studies (48) from 50 to 70 years ago suggest that polio virus preferentially affects the reticular formation, posterior hypothalamus, thalamus, putamen, caudate, locus co-eruleus, and substantia nigra which may account for the late-onset fatigue and attention deficit (49–52). Interestingly, cortical involvement is relatively selective and preferentially involves the precentral gyrus and pre-motor areas. A more recent case report (53) and a retrospective analysis of formalin-fixed central nervous system (CNS) tissue of a small cohort of patients (33) arrived at a different conclusion. They identified no cerebral involvement at all, but selective spinal cord pathology affecting the anterior roots with dorsal root sparing. These studies detected enterovirus RNA in spinal cord only. There have also been rare reports of polio patients developing ALS with characteristic histopathological findings (54, 55). Compared to other motor neuron diseases (56), there is a striking paucity of brain (57) and spinal cord imaging studies in PPS (58). Magnetic resonance imaging (MRI) has been used to evaluate volumetric changes (59) and to correlate anatomical changes to post mortem findings (48). The main focus of existing brain imaging studies in PPS was to explore the substrate of fatigue. Multiple hyperintensities were identified in the reticular formation, putamen and medial lemniscus in the majority of PPS patients (48) which is consistent with previous post mortem studies (49–52). A large study of 118 participants compared the brain volume profile of 42 PPS patients, 49 multiple sclerosis patients and 27 controls, and no statistically significant volume reductions were identified in PPS (59). No association was identified between fatigue and brain volumes. The majority of existing studies are cross-sectional which provide limited insights into progressive longitudinal alterations (60). There is an ongoing longitudinal, case-control study to characterize spinal cord alterations in PPS (61).

### Diagnosis

Post-polio syndrome is a clinical diagnosis, supported by electrophysiological findings and possible mimics need to be reassuringly ruled out. An extensive work-up including laboratory tests, imaging studies, cerebrospinal fluid sampling, detailed electrophysiological evaluation, and muscle biopsies may be required to exclude alternative diagnoses. The diagnostic criteria for PPS was first proposed by Halstead in 1991 (62) and evolved over time to the current March of Dimes diagnostic criteria (63, 64) which include:

1. Prior paralytic poliomyelitis with evidence of motor neuron loss, as confirmed by history of the acute paralytic illness, signs of residual weakness and muscle atrophy on examination, or signs of denervation on EMG.
2. A period of partial or complete functional recovery after acute paralytic poliomyelitis, followed by an interval (usually 15 years or more) of stable neuromuscular function.
3. Gradual onset (rarely abrupt) progressive and persistent new muscle weakness or abnormal muscle fatigability (decreased endurance), with or without generalized fatigue, muscle atrophy, or muscle and joint pain. Onset may at times follow trauma, surgery, or a period of inactivity. Less commonly, bulbar dysfunction or respiratory weakness occurs.
4. Symptoms that persist for at least a year.
5. Exclusion of alternative neuromuscular, medical, and orthopedic problems as causes of symptoms.

PCR amplification of poliovirus RNA in the CSF is indicative of prior history of poliomyelitis (6, 7, 32) and the presence of pro-inflammatory cytokines may also be detected (39, 65). Proteomic CSF markers such as gelsolin, hemopexin, peptidylglycine alpha-amidating monooxygenase, glutathione synthetase, and kallikrein 6 have been proposed as diagnostic markers but supporting evidence from larger studies is lacking (4). On muscle biopsy, hypertrophic muscle fibers type I (66, 67), indicative of compensatory reinnervation and small angulated fibers, indicative of active denervation (19) may be observed. CSF sampling and muscle biopsy also allows the exclusion of other neuromuscular mimics. People with PPS typically undergo detailed spinal imaging to rule out alternative structural, neoplastic, compressive, or inflammatory spinal etiologies which could manifest in lower motor neuron dysfunction (58, 68–70). Electromyography (EMG) is an invaluable tool to assess suspected post-polio cases, as it allows the confirmation of a prior history of poliomyelitis while excluding differential diagnoses (71). A variety of EMG techniques have been used in post-polio research studies including single fiber EMG (SFEMG), high density surface EMG (HDsEMG) (72), and macro-EMG. Ongoing denervation can be detected on conventional EMG by the presence of fibrillation and fasciculation potentials and increased jitter on SFEMG in newly weakened muscles (73). Needle EMG can also readily detect sub-clinically affected muscles in PPS (74). EMG measures correlate well with muscle strength and endurance (75, 76). While EMG provides important insights, EMG measures don't differ significantly between those with PPS and stable polio (77) and thus EMG is not regarded as an electrodiagnostic tool to confirm PPS (73). PPS is therefore a clinical diagnosis supported by laboratory tests.

### The Spectrum of Clinical Manifestations

**Figure 1:**
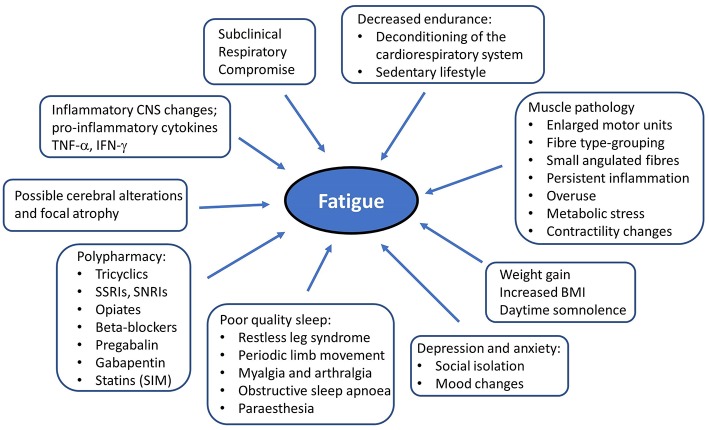
Putative factors in the etiology of generalized fatigue in post-polio syndrome. RLS, Restless leg syndrome; PLMS, periodic limb movement in sleep; CNS, Central nervous system.

Post-polio patients characteristically experience new onset muscle weakness, decreased endurance, muscle atrophy, myalgia, and fasciculations (78). Additional symptoms often include generalized fatigue, cold intolerance, dysarthria, dysphagia, and respiratory compromise (79, 80). New symptoms typically occur in previously affected areas but sub-clinically affected body regions can also get affected (74). Ambulatory difficulties often necessitate assistive devices, and may lead to increased fall risk (81). PPS is also associated with a wide range of non-motor symptoms. Frank sensory deficits may be detected and paraesthesias are often reported by PPS patients. Changes in sensory evoked potentials have been linked to cord atrophy on MRI (82). There have been consistent reports of cognitive deficits (83) in PPS including word finding difficulties (84), poor concentration, limited attention, memory impairment (85), and mood disturbances (86). The non-motor aspects of PPS are often under evaluated despite their considerable quality of life implications (87). Due to the combination of motor disability (88) and non-motor symptoms, many patients engage less in social activities (89) which may lead to social isolation. Generalized fatigue is one of the most distressing sequelae of PPS which is likely to be multifactorial due to muscle unit pathology, weight-gain, respiratory compromise, polypharmacy, and poor sleep (Figure 1). The identification of the key “fatigue-factors” in individual patients is indispensable for the effective pharmacological and non-pharmacological management of fatigue. Fatigue is thought to exhibit circadian variations throughout the day (90). Sleep disorders such as restless leg syndrome (RLS) (87, 91–94), sleep related breathing disturbances (95), obstructive sleep apnoea (OSA) (96), excessive daytime somnolence (EDS), and periodic limb movement in sleep (PLMS) (97) are not only often reported in PPS but they are likely to play an important role in the pathogenesis of fatigue in PPS (98, 99). Fatigue is thought to be more severe in PPS with RLS, and correlate to the severity of RLS (87). The simultaneous onset of RLS and PPS symptoms (91) and the positive response to pramipexole in an uncontrolled trial by Kumru et al. (93) have been interpreted as a pathophysiological link between RLS and PPS (98). The putative link between RLS and neuroimmunological alterations (100, 101) may also suggest shared pathophysiological processes between PPS and RLS (99). Furthermore, a higher incidence of cauda equina syndrome (102) and renal impairment (103) has also been reported in PPS but the association between these syndromes remains to be elucidated.

### Progression, Assessment, and Monitoring

The majority of longitudinal studies (14, 25, 104–107) detect progressive muscle weakness, which contributes to deteriorating gait performance (107) and declining mobility (105). Quantifying the rate of decline in PPS is challenging and no reliable functional predictors have been validated. Male gender is thought to be a negative prognostic indicator (108), but PPS is more common in females (12). Most PPS patients who participated in research studies have lived with PPS for over 13 years suggesting that PPS is a relatively slowly progressive condition. There have also been however sporadic reports of rapidly progressive and life-threatening forms of PPS (109), which raises the question of occasional misdiagnoses or a link between PPS and amyotrophic lateral sclerosis (ALS) (54). The severity of PPS-associate disability is typically evaluated clinically but a number of rating scales and questionnaires have been developed and validated for both clinical and research use. In addition to mobility and dexterity, these instruments evaluate the non-motor aspects of the condition such as fatigue, pain, sleeping disturbances, and mood (110). Clinical tests used to assess motor disability include the 6-min walking test (6MWT) (111) at self-preferred speed, the 2-min walking test (2MWT) at maximal speed (112), Timed-Up-and-Go test (TUG) (113), 10 meters walking test (10MWT), Sit-Stand-Sit test (SSS) (114). Muscle strength is typically appraised by manual muscle testing using the MRC scale, or more objectively using a dynamometer during maximal isokinetic and isometric voluntary contraction. Endurance is measured using isometric contraction peak torque, isometric endurance, tension time index (TTI) or recovery of torque after endurance test (76). Quantitative muscle mass assessment can be performed using ultrasound parameters such as muscle echo intensity and muscle thickness which are non-invasive tools for disease monitoring (115). The most commonly used instruments to assess non-motor domains include the Fatigue Severity Scale (FSS) (116), Fatigue Impact Scale (FIS), Piper Fatigue Scale (PFS), Short Fatigue Questionnaire (SFQ), Nottingham Health Profile (NHP), Physical activity scale for the elderly (PASE) (117), Polio Problem List (PPL), Visual analog scale (VAS) (118), Multidimensional Fatigue Inventory (MFI-20) (119), World Health Organization quality of life abbreviated scale (WHOQOL-BREF) (120), University of Washington Self-Efficacy Scale (UW-SES) (121), Sickness Impact Profile (SIP), 36-item Short Form Health Survey (SF-36) (112). Sleep disturbances (97) and respiratory function can be formally assessed through polysomnography and pulmonary function tests (PFT) (122, 123). RLS is typically diagnosed clinically (124) and most commonly evaluated using the validated international RLS rating scale (IRLS) (87, 93, 125). Maximal inspiratory and expiratory pressures (MIP and MEP), sniff nasal inspiratory pressure (SNIP) (126), and arterial blood gases are validate markers of respiratory function in PPS.

### Non-pharmacological Interventions

The effective management of the heterogeneous symptoms of PPS requires individualized care in a multidisciplinary setting (127). Expert input from physiotherapists, occupational therapists, speech and language therapists, respiratory physicians, podiatrists, psychologists, dieticians, pain specialists, social workers, nurse specialists, and orthotists are needed to meet the multifaceted care and support needs of PPS patients (128). Individualized lifestyle modifications and energy conservation strategies are indispensable in the effective management of PPS (129). PPS-specific training regimens alternating active intervals and rest have been developed to improve cardiorespiratory fitness, conserve energy during routine activities, and maintain independence (130). Isokinetic, isometric, resistance, and endurance training are thought to improve muscle strength and endurance without further muscle unit degeneration (131–140). Combining aerobic and flexibility training is also thought to improve QoL. Supervised training is advised in those with significant disability (141). Training in a warm environment may have longer lasting effects than training in colder temperatures (142). Patients with arthralgia may benefit from dynamic water exercises (143) as well as exercising in a group setting (144). Deconditioning of the cardiorespiratory system (145) may limit the effectiveness of aerobic training in PPS (146), therefore aerobic regimens must be carefully tailored to individual fitness levels (147). While some studies show improved endurance following mid- to high-intensity aerobic exercises (139, 140), a recent study (148) highlights that high-intensity aerobic exercise may not be beneficial in PPS patients with fatigue. Due to the heterogeneity of disability profiles in PPS, individualized training regimes and exercises that don't rely on anti-gravity strength are particularly important (148–150). Home-based arm ergometry for example is a well-tolerated and safe form of aerobic exercise (149, 150). Whole body vibration (WBV) has been proposed as an alternative to exercise in PPS (151) and improved mobility was reported in a small study (152), but no improvement was noted in muscle strength or gait performance (153). Orthoses are commonly prescribed for PPS patients to improve mobility and reduce pain. New powered-type Knee Ankle Foot Orthosis (KAFOs) offer limited benefits on gait symmetry or walking speeds but were shown to improve base support, swing time, stance-phase, and knee flexion during swing phase (154). The emergence of novel, light-weight materials such as carbon fiber (155) and the biomechanical analysis of individual walking patterns have helped to optimize orthosis-design for patients. The use of MIG3 Bioceramics fabrics for example had beneficial effects on pain and periodic limb movement (156). Other lifestyle modification such as weight loss, smoking cessation, increased physical activity, and modification to daily activities have all been beneficial to patients with PPS (22). There are sporadic reports that anodal transcranial direct current stimulation (tDCS) of premotor regions (157), repetitive transcranial magnetic stimulation (rTMS) of the left prefrontal cortex (158) and static magnetic fields (159) may ameliorate fatigue, improve sleep, reduce pain, and even improve motor functions in PPS, but these studies have not been replicated. PPS patients with bulbar involvement require expert phonatory and swallowing assessments by a speech-and-language therapist (160) and careful follow-up. Instrumental modalities such as ultrasonography and videofluoroscopy (161) and clinical instruments (162) can be used to detect progressive bulbar dysfunction and appraise the risk aspiration. Compensatory swallowing techniques, dietician input for food consistency alterations, individualized speech therapy, and laryngeal muscle training may be helpful in PPS patients with bulbar involvement (163). PPS patients who suffer from respiratory compromise and sleep related breathing disorders benefit from lung volume recruitment (LVR) (164) and non-invasive ventilation (NIV) such as Bi-PAP (165) or nasal intermittent positive-pressure ventilators (NIPPV) (166). Invasive ventilatory support with a tracheostomy is seldom required in PPS (167).

Addressing the non-physical aspects of PPS; mitigating psychological responses, emotional reactions, frustration, and fear of falling are equally important aspects of multidisciplinary care (168). Despite its positive effects on self-esteem (169), cognitive behavioral therapy (CBT) is not superior to standard multidisciplinary care in the treatment of fatigue (170–172). Psychotherapy is primarily aimed at reducing anxiety, improving depressive symptoms (173), alleviating pain (174, 175), and enhancing subjective well-being (176). Hope-oriented psychotherapy and encouraging participation in work (177) promote resilience in polio survivors and is associated with improved social functioning (178), satisfaction with social roles, improved quality of life, and superior mental health (179). Peer-support groups are also instrumental in buffering the impact of a functional impairment on psychosocial well-being (180). Furthermore, a reduction of physical demands at work and ergonomic adaptations at the workplace not only help PPS patients to maintain their occupational activities but enjoy their work (181). Rehabilitation nurses also play an important role in the setting of realistic health goals, encouraging resiliency, and providing emotional support (182).

### Pharmacological Trials

Several randomized controlled clinical trials (RCT) were conducted in PPS (Table 1). High-dose prednisone (183), amantadine (184), and modafinil (187, 188) showed no superiority to placebo in the management of fatigue. Prednisone therapy, showed a short-lived improvement in muscular strength but no meaningful functional improvement (183). The evidence for the benefit of pyridostigmine therapy remains conflicting. Some studies (185) identified no benefit on muscle function while others reported a slight improvement in walking performance (186). Co-enzyme Q10 supplements are thought to have no effect on muscle strength, endurance or fatigue in PPS (189, 190). A small RCT of lamotrigine, demonstrated improvements in VAS, NHP, and FSS suggesting that it may be beneficial to treat pain and fatigue and improve quality of life (191). Given the inflammatory and autoimmune hypothesis of PPS pathogenesis, intravenous immunoglobulin has been extensively investigated for its potential therapeutic effects. Its benefit with regards to pain, muscle strength, physical functioning, and quality of life is inconsistent. Improved pain control and overall vitality (192, 196) seem to be the main benefit of intravenous immunoglobulin (IVIg) treatment. Two small uncontrolled trials (38, 194) and two larger RCTs (40, 65) arrived to similar conclusions with regards to pain control and improvement in serum and CSF inflammatory markers. The main indicators for response to IVIg include severe pain, fatigue, <65 years of age, and paresis mainly affecting the lower extremities (194, 195, 198). Studies are somewhat conflicting on its effect on muscle strength (65, 193). These findings however encourage further large RCTs to establish the target PPS cohort for IVIg treatment, treatment intervals, and dose optimisation. A single-center, double-blind RCT trial of L-citrulline (197) is currently underway to investigate its effect on muscle metabolism and function. It is at clinical phase IIa and has proven to be of beneficial in muscular dystrophies in improving endurance in both aerobic and anaerobic exercise. The symptomatic management of non-motor symptoms in PPS also has considerable quality of life benefits. Restless leg syndrome in PPS often responds to dopamine agonists such as pramipexole (93, 199). The use of analgesics and antidepressants such as amitryptiline, duloxetine, and codeine may decrease physical discomfort and improve mood but need careful monitoring as they may worsen fatigue and lead to poor concentration. Adverse reactions to certain anesthetic agents are well-documented in PPS. Post-anesthesia fatigue, somnolence, and weakness are well-recognized, and fatal outcomes due to respiratory arrest have also been reported (200, 201). The diagnosis of PPS needs to be carefully discussed with the anaesthesiologists, so the appropriate muscle relaxants and anesthetics can be used, and patients should be advised of the possibility of a prolonged post-operative phase (202).

**Table 1:** Pharmaceutical and non-pharmaceutical clinical trials in post-polio syndrome; study characteristics and key outcomes.

**References**	**Study Design/selection criteria of PPS patients**	**Number of follow-up time points**	**Follow-up interval (months)**	**Number of participants receiving drug/placebo**	**Assessment tools used**	**Key study findings**
**PREDNISONE**						
Dinsmore et al. (183)	RDBPC/U	3	3	7/7	MRC scale, MVIC using electronic strain gauge tensiometer, fatigue on a 0–3 scale	- Short-lived improvement in muscle strength - No improvement in fatigue - Not recommended
**AMANTADINE**						
Stein et al. (184)	RDBPC/S (fatigue)	2	2	10/13	FSS, VAS-F, MMPI, BDI, somatization scale, reaction time evaluation	- Not superior to placebo for fatigue
**PYRIDOSTIGMINE**						
Trojan et al. (185)	RDBPC/S(fatigue/muscle weakness)	6	at 6 weeks, 10 weeks, and 6 months	43/42	SF-36, modified TQNE, MVIC by electronic strain gauge, Hare Fatigue Symptom Scale, FSS, IGF-1 serum levels	- Very weak muscles became slightly stronger - IGF-1 increased in compliant patients - No clear benefits on QoL, muscle strength, and fatigue
Horemans et al. (186)	RDBPC/S (fatigue and muscle weakness)	5	0.75	31/31	NHP, FSS, 2MWT at comfortable pace, time to walk 75 m at fastest speed, ambulatory activity monitor, MVC by chair dynamometer, MVA by interpolated stimulation; muscle fatigability by sEMG during 30 s sustained isometric contraction at 40% of MVC, NMJ defects by jitter on S-SFEMG	- No significant effects on fatigue - Significant effects on walking distance - Little effects on walking duration, muscle strength, MVA - Limited benefits in physical performance
**MODAFINIL**						
Chan et al. (187)	RDBPC cross-over/S (fatigue)	12	0.25	7/7 Cross-over 7/7	PFS, ESS, aural digit spans, reaction time	- Not effective in fatigue
Vasconcelos et al. (188)	RDBPC cross-over/ S (fatigue)	2	1.5	18/18 Cross-over 18/15	FSS, VAS-F, FIS; SF-36	- Not superior to placebo in fatigue and QoL improvement
**CO-ENZYME Q10**						
Skough et al. (189)	Parallel RDBPC/S(ability to perform resistance training)	2	3	7/7	Sit-stand-sit test (SSS); Timed up and go (TUG) test, 6MWT, dynamometer, bloods for CK, LD	- No change in CK or LD - No additional effects of the Co-enzyme Q10 supplementation during resistance training
Peel et al. (190)	Parallel RDBPC/S (fatigue)	2	2	54/49	MAF (revised Piper Fatigue Scale), FSS	- Not effective in fatigue
**LAMOTRIGINE**						
On et al. (191)	RDBPC/S (ambulatory with lower limb involvement only)	3	0.5	15/15	VAS, NHP, FSS	- Superior to placebo for pain, fatigue, and QoL as detected in VAS, NHP, FSS
**INTRAVENOUS IMMUNOGLOBULIN (IVIg)**						
Gonzalez et al. (38)	Controlled open-label/U	2	1.5-2	16PPS; 26OND/0	CSF for CSF-MC, PB for PBMC, real-time quantitative RT-PCR for relative quantitation of mRNA	- Significant decrease of CSF-MC expression of TNF-α and IFN-γ not seen in PBMC expression of cytokines
Kaponides et al. (192)	Uncontrolled open-label/S (ambulatory, BMI <28)	3	at 2 and 6 months	14/0	Dynamic dynamometer, 6MWT, SF-36	- No significant effect on muscle strength and physical performance
Gonzalez et al. (193)	RDBPC/U	2	3	67/68	Dynamometer, SF-36, 6MWT, TUG, PASE, sway, sleep quality, VAS, MFI-20	- Positive changes in muscle strength, physical activity, and those with significant pain - No change on QoL, fatigue sleep quality, “better” limb muscles or mild pain
Farbu et al. (40)	RDBPC/U	5	3	10/10	MAF (revised Piper Fatigue Scale), FSS, CSF, and PB for expression of cytokines (TNF-α, IFN-γ, IL-6, IL-1β, IFN-β, IL-10) using ELISA	- Positive effects on pain after 3 months - No effects on muscle strength and fatigue - TNF-α increased in CSF
Werhagen et al. (194)	Uncontrolled open-label/S (pain)	2	6	45/0	Neurological examination, sensory testing, soft tissue palpation, and joint assessment, VAS, pain classified according to IASP	- Better results on pain in younger, those with more pronounced paresis, had acute polio <10 yo
Östlund et al. (195)	Uncontrolled open-label/S(fatigue, muscle weakness)	2	6	113/0	SF-36, PASE, VAS	- Likely responders include those with pain intensity above VAS of 20 mm, younger than 65 yo, and paresis in lower extremities
Gonzalez et al. (65)	RDBPC and controlled quantitative cytokine study/U	2	12	CSE: 20/21 CAS: 20/30	CSE: SF-36, 6MWT, VAS CAS: CSF and PB for cytokines (TNF, IL-23, IFN-γ, TGF-β, IL-10, IL-13) using RT-PCR	- Improvement in QoL but not in pain and walking ability compared to placebo - Decline in CSF IFN-γ and IL-23, TNF, and increase in IL-10 and IL-13 - No changes in PB cytokine levels
Bertolasi et al. (196)	RDBPC/U	3	2	24/26	SF-36, MRC scale, dynamometer, 6MWT, VAS, 101-PNR, FSS	- Improvement in QoL; mental activity subscale - No effects on gait, muscle strength, fatigue, and pain
**L-CITRULLINE**						
Schmidt et al. (197)	RDBPC/U	5	6	15/15	6MWT, MFM scale, qMRI, MRS, bloods for muscle necrosis (CK), oxidative stress (8OHDG, 4-HNE), nitrosative stress(nitrotyrosine, cGMP), mitochondrial-related	- Ongoing clinical trial
					genes (*Citratsynthase, Cytochrome C oxidase subunit 1, Succinate dehydrogenase subunit A)*, QMT using HHD, SIPP,IBM-FRS,WHOQOL-BREF	
**RESPIRATORY SUPPORT**						
Kaminska et al. (164)	Feasibility/S(restrictive respiratory defects)	2	3	7ALS, 7PPS, 5MD	SF-36, SIP, standard spirometry (FVC, FVC% predicted, LIC, LIC-FVC difference, PCF, MIP, MEP)	- LVR Feasible - Encouraging effects on respiratory mechanics - LIC increased
Gillis-Haegerstrand et al. (165)	Randomized comparative/S(using VCV)	2	30 min	8	BP, oxygen saturation, ABG, indirect calorimetry (SaO_2_, VO_2_, VCO_2_, REE, RQ, RR, IPAP)	- BiPAP PSV decreases oxygen cost of breathing in PPS with respiratory failure without decreasing ventilation efficiency. - Significant PaCO_2_ decrease using this ventilation modality. - Maintains adequate ventilation in PPS patient with resp. failure
Barle et al. (167)	Comparative /S (nocturnal invasive CMV)	7	30 min	9	BP, oxygen saturation, ABG, indirect calorimetry (SaO_2_, VO_2_, VCO_2_, REE, RQ, MV,RR, IPAP)	- Invasive BiPAP reduces oxygen cost of breathing in long-standing tracheotomized PPS compared to CMV.
**EXERCISE PROGRAM**						
Murray et al. (149)	Assessor blinded rCT/U	2	2 months	26/29	6-MAT, PASIPD, 6MWT, FSS, SF-MPQ-2, QMA, exercise log	- Home-based ergometry is a well-tolerated form of aerobic exercise - No improvement of physical fitness, fatigue, activity - Slight decrease in BP in interventional group
**PRAMIPEXOLE**						
Kumru et al. (93)	Uncontrolled open label/U	3	At 0, 2 months and 6 months	16/0	RLS severity scale	- Significant decrease of RLS severity detected on RLS rating scale - Maintenance of improvement of RLS with pramipexole at 6 months follow-up

*rCT, randomized controlled trial; S, selected (i.e., fatigued); U, unselected; RDBPC, Randomized double-blind placebo controlled*.

## Conclusions

Despite being one of the most devastating neurodegenerative conditions in the world, surprisingly limited research is undertaken in post-polio syndrome. Its pathogenesis remains elusive, no sensitive diagnostic tools have been developed, and validated prognostic and monitoring markers are lacking. Non-motor symptoms of PPS have considerable quality of life implications and are notoriously challenging to manage. The etiology of fatigue in PPS is yet to be elucidated and successful individualized management strategies are needed to maintain mobility, independence, and patient autonomy. There is striking a paucity of neuroimaging studies in PPS that could provide anatomical insights into the substrate of extra-motor symptoms. Ultimately, the characterization of PPS-associated pathology may help research efforts in other motor neuron diseases.

## Author Contributions

The manuscript was drafted by SL and PB. The manuscript was edited, adjusted, and reviewed for intellectual content by RC, EF, DM, and OH.

### Conflict of Interest Statement

The authors declare that the research was conducted in the absence of any commercial or financial relationships that could be construed as a potential conflict of interest.

## Abbreviations

101-PNR: 101- point numeric rating
10MWT: 10-meter walk test
2MWT: 2-minute walk test
6MWT: 6-minute walk test
ALS: Amyotrophic lateral sclerosis
BDI: Beck depression inventory
BiPAP: Bilevel positive airway pressure
CAS: cytokine analysis study
CBT: Cognitive behavioral therapy
CK: Creatine kinase
CMAP: Compound muscle action potential
CMV: Controlled mechanical ventilation
CSE: Clinical study extension
CSF: Cerebrospinal fluid
CSF-MC: cerebrospinal fluid mononuclear cells
ELISA: Enzyme-linked immunosorbent assay
EMG: Electromyography
ESS: Epworth sleepiness scale
FIS: Fatigue impact scale
FSS: Fatigue severity scale
FVC: forced vital capacity
HDsEMG: High density surface electromyography
HHD: hand-held dynamometry
IASP: International Association for the Study of Pain
IBM-FRS: Inclusion body myositis functional rating scale
IPAP: inspiratory positive airway pressure
KAFO: Knee ankle foot orthosis
LIC: lung insufflation capacity
LVR: Lung volume recruitment
MAF: Multidimensional assessment of fatigue
MD: Myotonic dystrophy
MEP: Maximal expiratory pressure
MFI-20: Multidimensional functional inventory
MFM scale: Motor function measurement scale
MIP: Maximal inspiratory pressure
MMPI: Minnesota multiphasic personality inventory
MRC: Medical Research Council Scale for muscle strength
MRI: Magnetic resonance imaging
MRS: Magnetic resonance spectroscopy
MUAP: Motor unit action potential
MV: Minute ventilation
MVA: Maximal voluntary activation
MVC: Maximal voluntary contraction
MVIC: Maximal isometric voluntary contraction
NHP: Nottingham health profile
NIPPV: Nasal intermittent positive pressure ventilation
NIV: Non-invasive ventilation
OSA: Obstructive sleep apnea
PASE: Physical activity of the elderly
PBMC: peripheral blood mononuclear cells
PCF: unassisted peak cough flow
PFS: Piper fatigue scale
PFT: Pulmonary function test
PLMS: Periodic limb movements of sleep
PPL: Polio problem list
PPS: Post-polio syndrome
PV: Polio virus
qMRI: quantitative magnetic resonance imaging
QMT: Quantitative motor test
rCT: randomized controlled trial
RDBPC: Randomized double-blind placebo controlled
REE: resting energy expenditure
RLS: Restless leg syndrome
RNA: Ribonucleic acid
RQ: respiratory quotient
RR: respiratory rate
RT-PCR: Reverse transcription polymerase chain reaction
rTMS: Repetitive transcranial magnetic stimulation
S-SFEMG: Single fiber electromyography stimulation
SF-36: 36-item short form survey
SFEMG: Single fiber electromyography
SFQ: Short fatigue questionnaire
SIP: Sickness impact profile
SIPP: Self-reported impairments in persons with late effects of polio
*SMN* gene: Survival motor neuron gene
SNIP: Sniff nasal inspiratory pressure
SSS test: Sit-stand-sit test
tDCS: Transcranial direct current stimulation
TQNE: Turf's quantitative neuromuscular examination
TUG test: Timed-Up-and-Go test
UW-SES: University of Washington self-efficacy scale
VAS: Visual analog scale
VAS-F: Visual analog scale for fatigue
VCO_2_: carbon dioxide production
VO_2_: oxygen consumption
WBV: Whole body vibration
WHOQOL-BREF: World Health Organization quality of life abbreviated scale.
